# Tenovin-6-mediated inhibition of SIRT1/2 induces apoptosis in acute lymphoblastic leukemia (ALL) cells and eliminates ALL stem/progenitor cells

**DOI:** 10.1186/s12885-015-1282-1

**Published:** 2015-04-07

**Authors:** Yanli Jin, Qi Cao, Chun Chen, Xin Du, Bei Jin, Jingxuan Pan

**Affiliations:** 1 Department of Pathophysiology, Zhongshan School of Medicine, Sun Yat-sen University, Guangzhou, 510080 People’s Republic of China; 2 Department of Pediatrics, Sun Yat-sen Memorial Hospital, Sun Yat-sen University, Guangzhou, 510000 People’s Republic of China; 3 Department of Hematology, Guangdong Provincial People’s Hospital, Guangzhou, 510080 People’s Republic of China; 4 State Key Laboratory of Ophthalmology, Zhongshan Ophthalmic Center, Sun Yat-sen University, Guangzhou, 510060 People’s Republic of China; 5 Collaborative Innovation Center for Cancer Medicine, State Key Laboratory of Oncology in South China, Sun Yat-Sen University Cancer Center, Guangzhou, 510060 People’s Republic of China; 6 Present address: Institute of Tumor Pharmacology, Jinan University College of Pharmacy, 601 West Huangpu Blvd., Guangzhou, 510632 People’s Republic of China; 7 State Key Laboratory of Ophthalmology, Zhongshan Ophthalmic Center, Sun Yat-sen University, 54 South Xianlie Road, Guangzhou, 510060 People’s Republic of China

**Keywords:** Acute lymphoblastic leukemia, Targeted therapy, Epigenetics, SIRT1 inhibitor, Tenovin-6, Apoptosis, p53, β-catenin, Stem/progenitor cells

## Abstract

**Background:**

Acute lymphoblastic leukemia (ALL) is a heterogeneous group of malignant disorders derived from B- or T-cell lymphoid progenitor cells. ALL often is refractory to or relapses after treatment; thus, novel targeted therapy for ALL is urgently needed. In the present study, we initially found that the level of SIRT1, a class III histone deacetylase, was higher in primary ALL cells from patients than in peripheral blood mononuclear cells from healthy individuals. But it is not clear whether inhibition of SIRT1 by its selective small molecule inhibitor Tenovin-6 is effective against ALL cells.

**Methods:**

We tested the effect of Tenovin-6 on ALL cell lines (REH and NALM-6) and primary cells from 41 children with ALL and 2 adult patients with ALL. The effects of Tenovin-6 on cell viability were determined by MTS assay; colony-forming assays were determined by soft agar in ALL cell lines and methylcellulose medium in normal bone marrow cells and primary ALL blast cells; cell apoptosis and cell cycling were examined by flow cytometry; the signaling pathway was determined by Western blotting; ALL stem/progenitor cells were seperated by using MACS MicroBead kit.

**Results:**

The results showed that Tenovin-6 treatment activated p53, potently inhibited the growth of pre-B ALL cells and primary ALL cells, and sensitized ALL cells to frontline chemotherapeutic agents etoposide and cytarabine. Tenovin-6 induced apoptosis in REH and NALM-6 cells and primary ALL cells and diminished expression of Mcl-1 and X-linked inhibitor of apoptosis protein (XIAP) in such cells. Furthermore, inhibition of SIRT1 by Tenovin-6 inhibited the Wnt/β-catenin signaling pathway and eliminated ALL stem/progenitor (CD133 + CD19-) cells.

**Conclusion:**

Our results indicate that Tenovin-6 may be a promising targeted therapy for ALL and clinical trials are warranted to investigate its efficacy in ALL patients.

## Background

Acute lymphoblastic leukemia (ALL) is a heterogeneous group of malignant disorders derived from B- or T-cell lymphoid progenitor cells. ALL is ranked as the fifth most common childhood cancer and accounts for a large proportion of cancer-associated deaths in children every year [1]. Over the past 50 years, advances in chemotherapy regimens have increased the cure rate for children with newly diagnosed ALL in the developed world to approximately 85% [1]. However, the remaining approximately 15% of children with ALL are not expected to survive because of relapse [2]. The problems of relapse, morbidity, and mortality are even more pronounced in adult patients with ALL. Novel treatments are desperately needed in order to improve survival in patients with ALL that is refractory to treatment or relapses after an initial response.

ALL has been shown to be associated with genetic and epigenetic alterations [3], and progress in elucidating the pathogenesis of ALL has revealed a large number of potential targets for anticancer therapy. For example, the discovery that Bcr-Abl is expressed in approximately 30% of cases of ALL in adults has been successfully translated into treatment with small molecule tyrosine kinase inhibitors (e.g., imatinib and bosutinib) [4]. The ETV6-RUNX1 fusion gene is found in approximately 25% of cases of ALL in children [5]. Chatterton et al. reported that 325 genes were hypermethylated and downregulated and 45 genes were hypomethylated and upregulated in pediatric B-cell ALL [6]. Epigenetic alteration indicates that targeted therapy against ALL is promising. Excitingly, vorinostat, a pan-histone deacetylase inhibitor, and more recently romidepsin, a bicyclic pan-histone deacetylase inhibitor, have been approved by the US Food and Drug Administration for treatment of relapsed or refractory cutaneous T-cell lymphoma [7].

Reversible protein acetylation is an important posttranslational modification that regulates the function of histones and many other proteins [8]. Histone acetylation is mediated by histone acetyl transferases (e.g., p300, CBP, and p/CAF in mammalian cells), while acetyl groups are removed by histone deacetylases [9]. Recently, the histone deacetylase sirtuin 1 (SIRT1) has been shown to be important in leukemia. Sirtuin 1 (SIRT1) is a stress-response and chromatin-silencing factor belonging to the class III histone deacetylases family, which is involved in various nuclear events such as transcription, DNA replication, and DNA repair [10]. SIRT1 has been shown to inhibit the maturation of preadipocytes [11] and promote resistance to conventional chemotherapeutic agents [12,13]. Additionally, mammalian SIRT1 is a key regulator of cancer cell survival in the face of cellular stresses. SIRT1 and other sirtuins were found to regulate cell survival during stress through deacetylation of key cell cycle and apoptosis regulatory proteins, including p53 [14,15], Ku70 [16], and forkhead transcription factors [10]. Of importance, SIRT1 is highly overexpressed in several types of tumors [17]. Recently, SIRT1 has been demonstrated to promote Bcr-Abl-driven leukemogenesis and the survival of chronic myelogenous leukemia stem cells [18,19].

In the present study, we initially discovered that SIRT1 level was higher in primary ALL cells than in control cells. We then hypothesized that inhibition of SIRT1 by its specific small molecule inhibitor Tenovin-6 induces apoptosis in ALL cells by releasing the expression of tumor suppressor genes such as p53. We tested this hypothesis in ALL cell lines (REH and NALM-6) and in primary cells from 41 children with ALL and 2 adult patients with ALL. Our findings suggest that Tenovin-6 may be a promising agent for ALL therapy.

## Methods

### Reagents

Tenovin-6 was purchased from Cayman Chemical (Ann Arbor, MI). Antibodies against SIRT1 (H-300), p53 (DO-7), cyclin D1 (C-20), Mcl-1 (S-19), and proliferating cell nuclear antigen (PCNA) were from Santa Cruz Biotechnology (Santa Cruz, CA). Antibodies against PARP (clone 4C10-5), caspase-3, XIAP, and anti-CD19 conjugated with phycoerythrin were from BD Biosciences (San Jose, CA). Antibodies against K382-acetyl-p53 and c-Myc were from Cell Signaling Technology (Beverly, MA). Anti-SIRT2 was purchased from Atlas Antibodies. The CD133 MicroBead Kit including anti-CD133 conjugated with APC was from Miltenyi Biotec, Inc. (Shanghai, China). Anti-mouse immunoglobulin G and anti-rabbit immunoglobulin G horseradish peroxidase-conjugated secondary antibodies were from Pierce Biotechnology (Rockford, IL).

### Cell culture

REH and NALM-6 cells from American Type Culture Collection (Rockville, MD) were cultured in RPMI 1640 (Invitrogen, Shanghai) supplemented with fetal calf serum (FCS; Kibbutz Beit, Haemek, Israel) and 100 units/mL penicillin and streptomycin at 37°C in a humidified atmosphere of 95% air and 5% CO_2_.

### Primary cells from patients with ALL

Peripheral blood or bone marrow samples from 43 patients with ALL (Children with ALL, 41 cases; Adult patients with ALL, 2 cases), acute myelogenous leukemia (AML; 4 cases), Lymphoma (1 case), and 5 healthy adult donors were obtained from the Sun Yat-sen Memorial Hospital of Sun Yat-sen University and Guangdong Provincial People’s Hospital. This study was approved by the Sun Yat-sen University Ethics Committee according to institutional guidelines and the Declaration of Helsinki principles, and written informed consent to participate in this research and written informed consent to publish the resultant results were obtained from all the patients involved or their legal guardians for children under the age of 16. The clinical information for the 48 patients is in Table 1.

**Table 1:** **Clinical characteristic of patients with leukemia**

Patient	Age, years/sex	WBC count (×10^9^)	Diagnosis	Mutations	Initial or relapsed disease	IC_50_for Tenovin-6, μM
1	11/M	216.45	ALL-L2, B	BCR/ABL (+, 72%)	Initial	5.44
2	4/M	1.09	ALL-L2, B	Neg	Initial	14.65
3	3/F	4.27	ALL-L2, B	Neg	Initial	8.05
4	10.6/M	17.66	ALL-L2, B	Neg	Initial	4.7
5	0.6/M	23.16	ALL-L2, B	Neg	Initial	2.72
6	10/F	531	ALL-L2, T	Neg	Initial	7.49
7	2.3/F	14.97	ALL-L2, B	MLL (+, 82%)	Initial	7.15
8	10/M	22.98	ALL-L2, T	Neg	Initial	10.49
9	10.6/M	17.66	ALL-L2, B	Neg	Initial	2.03
10	2.4/F	24	ALL-L2, B	Neg	Relapsed	5.9
11	10/M	43.8	ALL-T	Neg	Relapsed	4.5
12	1.9/M	44.2	ALL-L2, B	Neg	Initial	3.03
13	3.5/F	5.2	AML-M0	Neg	Initial	8.15
14	1.6/F	5.1	AML-M7	Neg	Initial	3.08
15	3/M	4.27	ALL-L2, B	Neg	Initial	2.88
16	2/M	58.9	ALL-L1, B	unknown	Initial	4.03
17	13/M	5.66	ALL-L2, B	Neg	Relapsed	6.98
18	9.5/M	10.59	ALL-L2, B	Neg	Initial	7.21
19	0.7/M	43	ALL-L2, B	Neg	Initial	13.82
20	7/M	6.55	ALL-L2, B	Neg	Relapsed	17
21	0.2/M	54.83	ALL-L2, B	MLL (+, 86%)	Initial	4.83
22	0.5/M	208.85	ALL-L2, B	Neg	Initial	3.8
23	0.8/F	137.41	ALL-L2, B	MLL (+, 96%)	Initial	7.24
24	11/M	24.06	ALL-L2, B	Neg	Initial	3.91
25	11/M	56.8	ALL, mature B	Neg	Initial	4.26
26	2/F	281.31	ALL-L1, T	Neg	Initial	4.13
27	10/F	34.7	AML-M3b	PML-RARa (+, 35%)	Initial	7.83
28	3/F	8.07	ALL-L2, B	TEL/AML1 (+, 85%)	Initial	3.58
29	14.2/M	104	ALL-L2, B	BCR/ABL (+, 82%)	Initial	3.75
30	12/M	384	ALL-L2, B	Neg	Initial	8.16
31	4/M	2.01	ALL-L2, B	TEL/AML (+, 75%)	Initial	6.15
32	12/M	189	ALL-L2, T	Neg	Initial	7.07
33	1.5/F	4.7	ALL-L2, B	Neg	Initial	4.74
34	2/F	26.7	ALL	Neg	Initial	5.32
35	9/F	3.8	ALL-L2, B	Neg	Initial	12.48
36	4.2/M	17.4	ALL-L2, B	Neg	Initial	6.2
37	6/F	52.53	ALL-L2, B	TEL/AML (+)	Initial	3.35
38	8/M	8.95	ALL-T	Neg	Initial	4.31
39	8/M	34.3	ALL-L2, B	Neg	Initial	4.03
40	5/M	26.46	ALL-L2, B	Neg	Initial	12.56
41	9/M	3.29	ALL-L2, B	Neg	Initial	10.2
42	1/M	21.6	AML	Neg	Initial	14.75
43	12/M	82.79	ALL-L1, T	Neg	Initial	6.65
44	4/M	6.62	Lymphoma	Unknown	Initial	4.81
45	0.8/F	137.41	ALL-L2	MLL (+, 96%)	Initial	13.22
46	11/M	24.06	ALL-L2	Neg	Initial	16.38
47	55/F	62.75	ALL	Neg	Initial	ND
48	28/F	2.65	ALL	Neg	Initial	ND

AML, acute myelogenous leukemia; WBC, white blood cell; Neg, negative; ND, not detected.

Mononuclear cells were isolated by Histopaque gradient centrifugation (density 1.077; Sigma-Aldrich, Shanghai) [20-22]. Contaminating red cells were removed by incubation in 0.8% ammonium chloride solution for 10 min. After a washing, cells were suspended in RPMI 1640 medium supplemented with 10% FCS. All drug treatments started after the cells were precultured in fresh medium for 24 hours.

For separation of stem/progenitor cells of ALL, the mononuclear cells were mixed with MicroBeads conjugated to monoclonal anti-human CD133 antibodies (isotype: mouse IgG1, clone AC133) and loaded onto a MACS column with separator according to the instructions from Miltenyi Biotec Inc [20]. After removing from the magnetic field, the magnetically retained CD133+ cells were eluted as the positively selected cell fraction. The purity was examined with a flow cytometer after staining of CD133-APC.

### Cell viability assay

Cell viability was evaluated by MTS assay (CellTiter 96 AQueous One Solution reagent, Promega, Shanghai) as described previously [20-22]. The IC_50_ was determined by curve fitting of the dose–response curve.

### Colony-forming assays

#### Soft agar clonogenic assay in ALL cell lines

ALL cell lines were treated with Tenovin-6 or diluent (DMSO, control) for 24 hours, washed with PBS, and seeded in Iscove's medium containing 0.3% agar and 20% FCS in the absence of drug treatment [20-22].

### Colony-forming assay in normal bone marrow cells and primary ALL blast cells

The colony-forming capacity of normal bone marrow cells and primary ALL blast cells was analyzed by use of methylcellulose medium (Methocult H4434, Stem Cell Technologies) according to the manufacturer's instructions. Tenovin-6 was added to the initial cultures at a concentration of 1 μM to 10 μM. After 14 days of culture, the number of colony-forming units was evaluated under an inverted microscope according to standard criteria [20-22].

### Reverse transcription and quantitative real-time PCR

Total RNA from cultured cells was extracted using Trizol reagent (Invitrogen, Shanghai). Two micrograms of RNA was processed directly to cDNA by reverse transcription with SuperScript III following the manufacturer’s instructions (Invitrogen, Shanghai). PCR primers for each gene were designed using real-time PCR primer design; sequences used in this study were as follows: *p53,* forward *5’-*GTGGAAGGAAATTTGCGTGT-3’, reverse 5’-TGGTGGTACAGTCAGAGCCA-3’; *p21,* forward 5’-GACTCTCAGGGTCGAAAACGG-3’, reverse 5’-GCGGATTAGGGCTTCCTCTT-3’; *Nox*a, forward 5’-GCAAGAACGCTCAACCGAG-3’, reverse 5’-TTGAAGGAGTCCCCTCATGC-3’; *Puma,*forward 5’-ACCTCAACGCACAGTACGAG-3’, reverse 5’-CGGGTGCAGGCACCTAATTG’; *Bax*, forward 5’-GAACCATCATGGGCTGGACA’, reverse 5’-GCGTCCCAAAGTAGGAGAGG’; *c-myc,* forward 5’-CAGCGACTCTGAGGAGGAAC-3’, reverse 5’-TCGGTTGTTGCTGATCTGTC-3’; *cyclin-D1,* forward 5’-GCTGTGCATCTACACCGACA-3’, reverse 5’-CCACTTGAGCTTGTTCACCA-3’; *LEF1,* forward 5’-CGAATGTCGTTGCTGAGTGT-3’, reverse 5’-GCTGTCTTTCTTTCCGTGCT-3’; *18 s,* forward 5’-AAACGGCTACCACATCCAAG-3’, reverse 5’-CCTCCAATGGATCCTCGTTA-3’. We used SYBR Premix Ex Taq (Perfect Real-time; Takara Bio) for qRT-PCR with Applied Biosystems 7500 Real-time PCR System (Applied Biosystems) according to the manufacturer’s instructions. The specificity of PCR products was checked on agarose gel. Expression levels of 18S rRNA were used as an endogenous reference.

### Western blotting analysis

Whole cell lysates prepared in RIPA (radioimmunoprecipitation) assay buffer (1 × PBS, 1% NP-40, 0.5% sodium deoxycholate, 0.1% SDS, 0.1 mg/ml phenylmethanesulfonyl fluoride, 20 mM sodium fluoride, 0.2 mM sodium orthovanadate, and Complete Protease Inhibitor Mix, one tablet per 50 ml) [20-22]. Cytoplasmic and nuclear fractions were prepared as described previously [20-22]. Protein samples were separated on SDS-PAGE gel and transferred to nitrocellulose membranes, which were then incubated with the primary antibodies. After incubation with appropriate secondary antibodies, the immunoblots were developed using SuperSignal Western blotting kits (Pierce Biotechnology) and exposed to X-ray film according to the manufacturer’s protocol. Western blots were stripped between hybridizations with stripping buffer [10 mM Tris–HCl (pH 2.3) and 150 mM NaCl].

### Flow cytometry analysis of cell cycle

After drug treatment, cells were collected and fixed overnight in 66% cold ethanol at −20°C. The cells were then washed twice in cold PBS and labeled with propidium iodide for 1 hour in the dark. Cell cycle distribution was determined by use of a FACSCalibur flow cytometer with CellQuest software [20-22].

### Measurement of apoptosis

Apoptosis was evaluated with an AnnexinV-fluoroisothiocyanate apoptosis detection kit according to the instructions of the manufacturer (Sigma-Aldrich, Shanghai) and analyzed with use of a FACSCalibur flow cytometer and CellQuest software as previously described [20-22].

### Electrophoretic mobility shift assay

The WT-TCF probe was prepared by annealing 5’-TGCCGGGCTTTGATCTTTG-3’ and 5’-AGCAAAGATCAAAGCCCGG-3’ deoxyoligonucleotides [23]. Double-stranded probes were end-labeled using biotin. EMSA was performed with use of the Light Shift Chemiluminescent EMSA kit (Pierce Biotechnology) according to the manufacturer's instructions [20].

### Statistical analysis

Data from all the experiments are expressed as mean ± 95% CI unless otherwise stated. GraphPad Prism 5.0 software (GraphPad Software, San Diego, CA) was used for statistical analysis. Comparisons among multiple groups involved one-way ANOVA with post-hoc intergroup comparison with the Tukey test. *P* < 0.05 was considered statistically significant.

## Results

### SIRT1/2 are increased in primary leukemia cells from patients with ALL and in ALL cell lines

**Figure 1:**
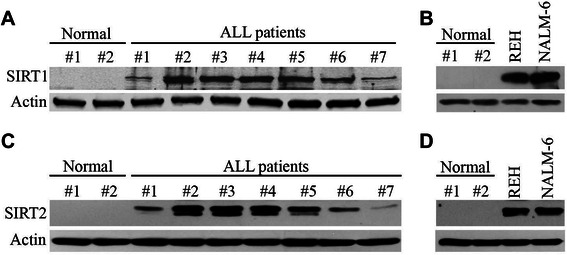
**The levels of SIRT1 and SIRT2 are increased in primary malignant cells from patients with ALL.** Western blotting analysis of SIRT1 in whole cell lysates from 7 patients with ALL **(A)** and REH and NALM-6 ALL cells and 2 healthy individuals **(B)**. Western blotting analysis of SIRT2 in whole cell lysates from 7 patients with ALL **(C)** and REH and NALM-6 ALL cells and 2 healthy individuals **(D).**

We first examined whether SIRT1 was increased in primary leukemia cells from patients with ALL. By using Western blotting, we examined the levels of SIRT1 in whole cell lysates of mononuclear cells from peripheral blood or bone marrow from 7 patients with ALL and 2 healthy individuals. The results revealed that the level of SIRT1 protein was higher in the whole cell lysates from the patients with ALL than in the whole cell lysates from the healthy individuals (Figure 1A). SIRT1 was also highly expressed in REH and NALM-6 ALL cells (Figure 1B). We also determined the levels of SIRT2 in ALL cells with Western blotting analysis. The expression of SIRT2 was much higher in the primary leukemia cells from ALL patients and in ALL cell lines than normal cells (Figure 1C and D).

### Tenovin-6-mediated inhibition of SIRT1/2 leads to hyperacetylation of p53 in ALL cells

**Figure 2:**
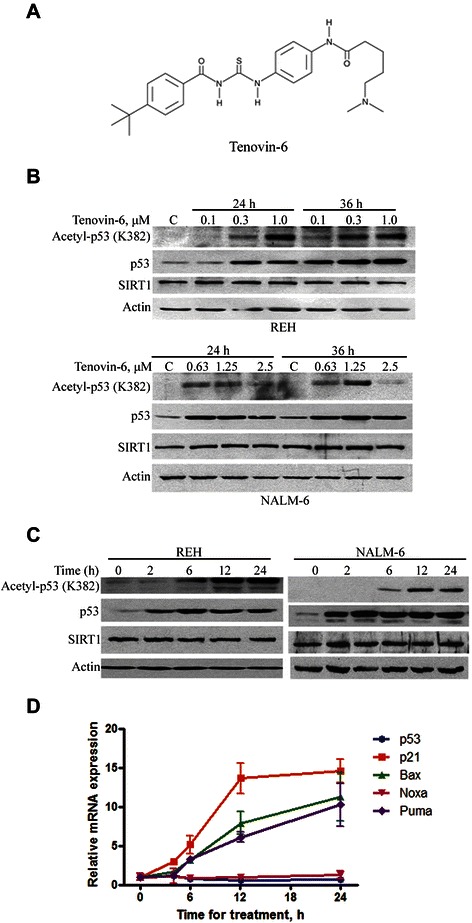
**Tenovin-6 induces activation of p53 in ALL cells. A**, Molecular structure of SIRT1/2 inhibitor Tenovin-6. **B**, REH and NALM-6 ALL cells were treated with increasing concentrations of Tenovin-6 for 24 and 36 hours. Acetylated p53, total p53 protein, and SIRT1 were detected by Western blotting analysis with the indicated antibodies. **C**, REH and NALM-6 cells were exposed to 1 μM Tenovin-6 for the indicated times, and acetylated p53, total p53 protein, and SIRT1 were detected by Western blotting. **D**, REH cells were treated with 1 μM Tenovin-6 for the indicated times, and mRNA levels of p53 and its targets-genes p21, Puma, Noxa and Bax were examined by real-time PCR. 18 s rRNA was used as an internal reference.

Tenovin-6 (molecular structure, Figure 2A) has been shown to inhibit the deacetylation activity of SIRT1 and SIRT2 [24]. We next examined the effect of Tenovin-6-mediated SIRT1/2 inhibition on the acetylation status of p53, an important substrate of SIRT1. Toward this end, REH and NALM-6 cells were exposed to increasing concentrations of Tenovin-6 for 24 and 36 hours. Western blotting of whole cell lysates revealed the anticipated increase in total and hyperacetylated p53 protein (Figure 2B). A time-course study showed that Tenovin-6 at a concentration as low as 1 μM elevated the total protein level of p53 within 2 hours, and that this increase was followed by a time-dependent increase in the acetylation level of p53 in both REH and NALM-6 cells (Figure 2C).

To evaluate whether Tenovin-6 increased p53 activation, we examined the transcription of known p53 target genes p21, Puma, Noxa and Bax. In accordance with the increased acetylation level of p53 after Tenovin-6 treatment, quantitative reverse transcriptase-polymerase chain reaction (qRT-PCR) analysis showed that Tenovin-6 appreciably promoted the transcription of p21, Puma, and Bax, but Noxa without changing the mRNA level of p53(Figure 2D).

### Tenovin-6 inhibits the growth of ALL cells

**Figure 3:**
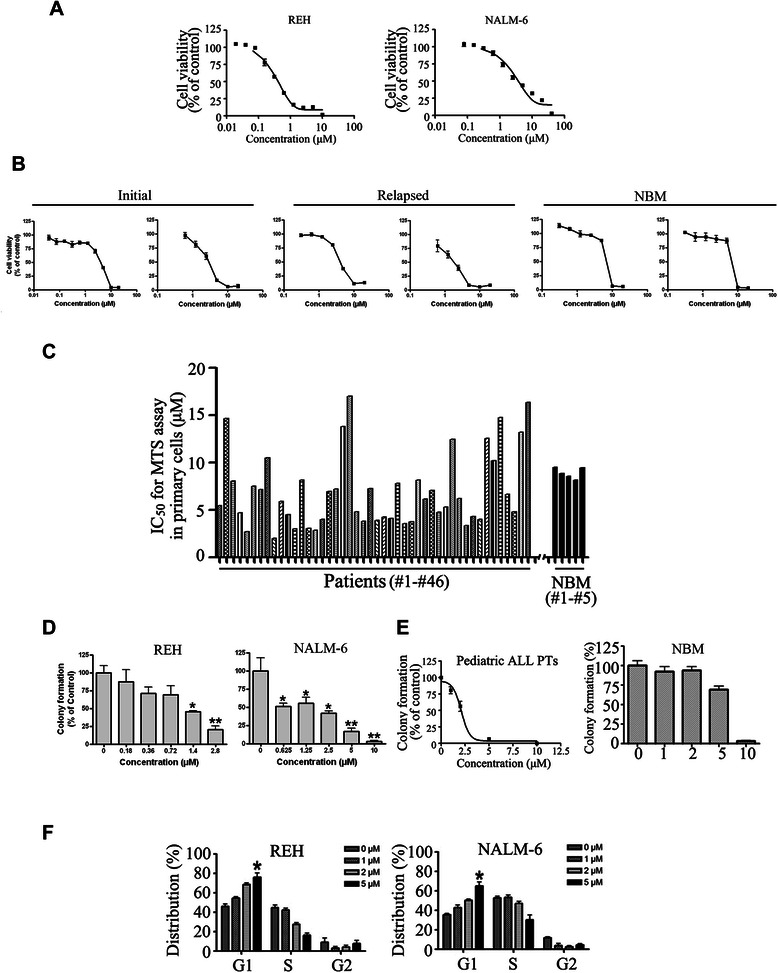
**Tenovin-6 inhibits the growth of ALL cells. A**, REH and NALM-6 ALL cells were exposed to Tenovin-6 for 72 hours. Cell viability (percentage relative to control) was determined by MTS assay. Tenovin-6 dose–response curves are shown. **B & C**, Mononuclear cells from peripheral blood of 46 children with primary (“Initial”) or relapsed ALL and from bone marrow of 5 healthy individuals (normal bone marrow; NBM) were exposed to increasing concentrations of Tenovin-6 and then subjected to MTS assay. Representative dose–response curves (B) and IC_50_ values of Tenovin-6 for all patients and healthy individuals (C) are shown. **D**, Tenovin-6 inhibited the clonogenicity of ALL cells. REH and NALM-6 cells were seeded in soft agar with the indicated concentrations of Tenovin-6 for 14 days, and then colony-forming units were counted. * P < 0.05, ** P <0.01, one-way ANOVA, *post hoc* comparisons, Tukey’s test; error bars represent 95% CIs. **E**, Colony-forming capacity of primary ALL bone marrow cells from 4 children with ALL and 3 normal bone marrow cells were evaluated by using methylcellulose medium with the indicated concentration of Tenovin-6. A representative curve is shown. **F**, Cell cycle distributions in REH and NALM-6 cells after exposure to increasing concentrations of Tenovin-6. * P < 0.05 compared with control.

The effect of Tenovin-6 on the viability of ALL cells was first examined by MTS assay. Tenovin-6 dose-dependently inhibited the growth of ALL cells; the drug concentrations resulting in 50% inhibition of cell growth (IC_50_ values) were 0.36 μM and 2.5 μM in REH and NALM-6 cells, respectively (Figure 3A). Because of these findings, we were curious to see whether Tenovin-6 also inhibited the growth of primary cells from patients with ALL. Peripheral blood mononuclear cells isolated from 43 patients with ALL (Table 1) and normal bone marrow cells from 5 healthy individuals were exposed to escalating concentrations of Tenovin-6 for 72 hours and then subjected to MTS assay for measurement of cell viability. The results showed that Tenovin-6 inhibited the growth of primary ALL cells in a dose-dependent manner; median IC_50_ values were 6.2 μM (range, approximately 2.03-17 μM) for ALL cells (Figure 3B & C and Table 1) and approximately 10 μM in normal bone marrow cells (Figure 3B & C). Of note, 4 of the 43 patients with ALL whose cells were treated with Tenovin-6 had relapsed ALL.

We next measured the effect of Tenovin-6 on the anchorage-independent growth of ALL cells REH and NALM-6 in soft agar culture. Tenovin-6 dose-dependently inhibited the number of surviving clonogenic ALL cells, with IC_50_ values of approximately 1.0 μM to 2.0 μM (Figure 3D).

Because of the efficacy of Tenovin-6 in primary cells from patients with relapsed ALL, we examined the effect of Tenovin-6 on functionally defined ALL stem/progenitor cells by methylcellulose colony assay. The colony-forming ability of primary ALL cells was strikingly inhibited by Tenovin-6 in a dose-dependent manner, with a median IC_50_ value of 2.59 μM (n = 4; Figure 3E, *left*). In contrast, Tenovin-6 inhibited the colony-forming ability of normal bone marrow cells with a median IC_50_ value of 7.53 μM (n = 3, Figure 3E, *right*).

We also assessed whether Tenovin-6 disturbed the cell cycle distribution of ALL cells. As shown in Figure 3F, exposure of ALL cells to increasing concentrations of Tenovin-6 for 24 hours dramatically arrested the cells in G_1_ phase.

### Tenovin-6 induces apoptosis in ALL cell lines as well as primary ALL cells

**Figure 4:**
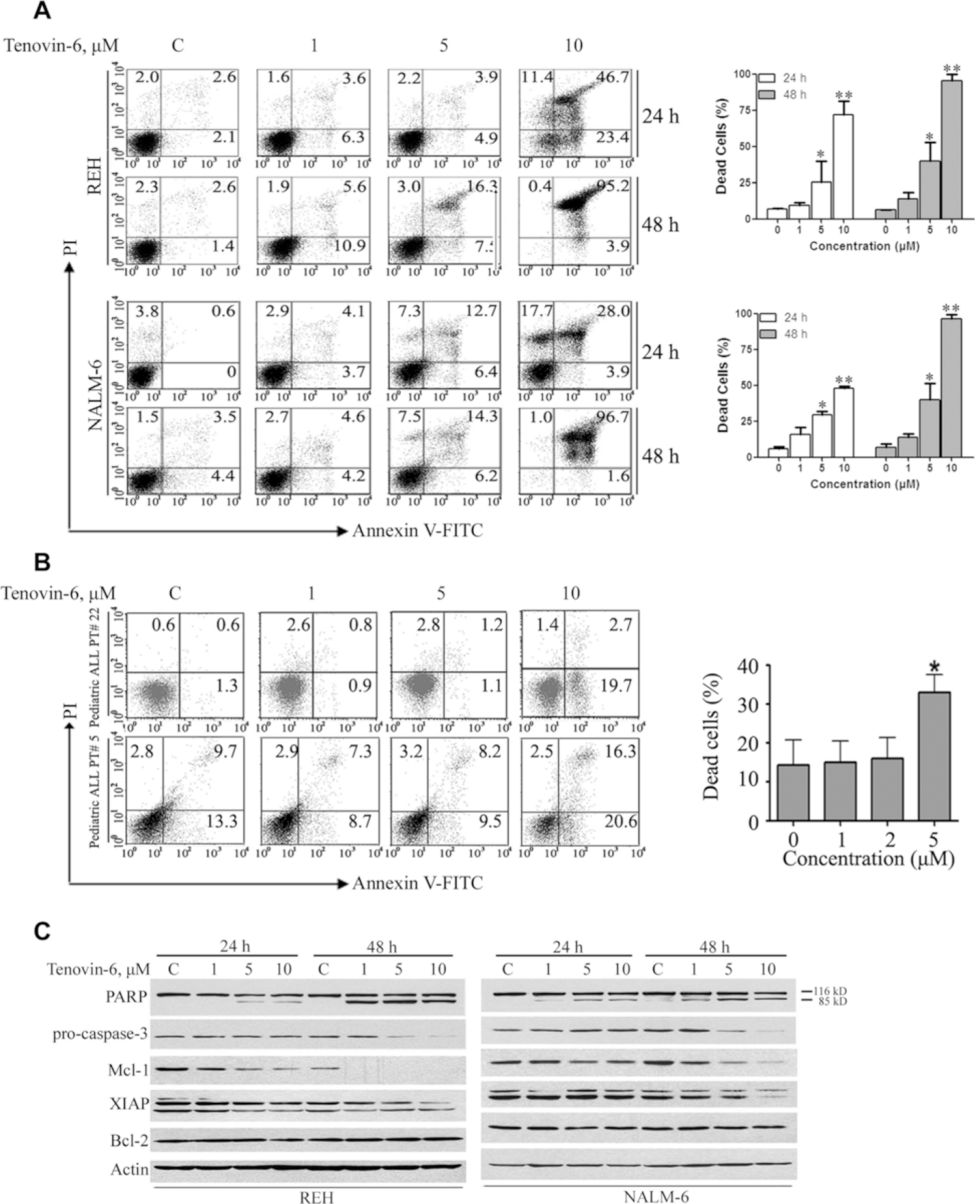
**Tenovin-6 induces apoptosis in ALL cells. A**, REH and NALM-6 cells were treated with increasing concentrations of Tenovin-6 for 24 or 48 hours. Apoptosis was determined by flow cytometry after Annexin V-FITC/propidium iodide staining. Representative histograms (*left*) and results for 3 independent experiments (*right*) are shown. **B**, Dead cells (including apoptotic and necrotic cells) in primary cells from children with ALL were examined by flow cytometry after treatment with Tenovin-6 for 48 hours. Histograms from 2 representative patients (*left*) and statistical analysis in 3 patients (*right*) are shown. * P < 0.05, ** P <0.01, one-way ANOVA, *post hoc* comparisons, Tukey’s test; error bars represent 95% CIs. C, REH and NALM-6 cells were treated with the indicated concentrations of Tenovin-6 for 24 or 48 hours. Time- and dose-dependent cleavage of PARP and levels of pro-caspase 3, Mcl-1, and XIAP were detected by Western blotting.

The impact of Tenovin-6 on apoptosis in ALL cells was detected by flow cytometry after Annexin V-fluorescein isothiocyanate (FITC)/propidium iodide staining. Exposure of REH and NALM-6 cells to increasing concentrations (range, approximately 1 to 10 μM) of Tenovin-6 resulted in massive apoptotic cell death (Figure 4A, *left*). Statistical analysis of cell death (including apoptotic and necrotic cells) induced by Tenovin-6 in REH and NALM-6 cells is presented in Figure 4A (*right*). Increased apoptosis was also detected in Tenovin-6-treated primary ALL cells from patients compared with untreated control cells (Figure 4B).

By Western blotting, we discovered that Tenovin-6 induced a dose- and time-dependent specific cleavage of poly(ADP-ribose) polymerase (PARP), a hallmark of apoptosis, and a decrease in pro-caspase-3, the precursor form of caspase-3, in REH and NALM-6 ALL cells, indicating onset of apoptosis (Figure 4C). Western blotting also revealed no change in the expression of Bcl-2 but a substantial decrease in XIAP and Mcl-1 with Tenovin-6 treatment (Figure 4C).

### Tenovin-6 sensitizes ALL cells to conventional chemotherapeutic agents

**Figure 5:**
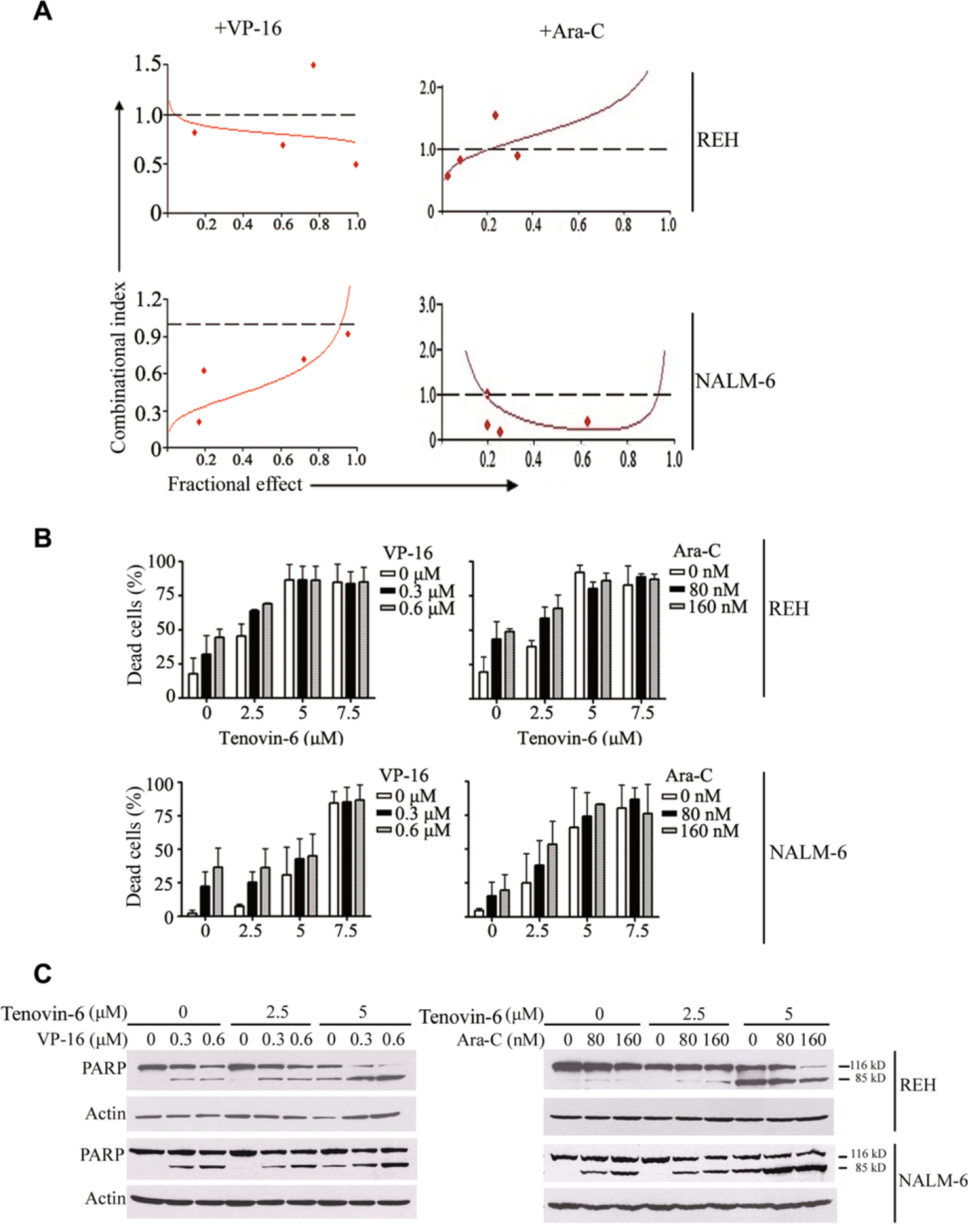
**Tenovin-6 sensitizes ALL cells to conventional chemotherapeutic agents. A**, Synergistic effect of the combination of Tenovin-6 and etoposide or cytarabine in ALL cells was assessed by MTS assay after incubation of ALL cells with a serial diluted mixture (at a fixed ratio) of the 2 drugs for 72 hours. CI is plotted against the fraction affected. A reference line is drawn at CI = 1. CI values of <1 indicated synergism between the 2 drugs. **B**, REH and NALM-6 cells were treated with the indicated concentrations of Tenovin-6 and etoposide or cytarabine for 48 hours. Apoptosis was determined by flow cytometry. The results shown are representative of 3 independent experiments. **C**, Apoptosis-inducing capacity of Tenovin-6 plus etoposide or cytarabine was examined by Western blotting for cleaved PARP in ALL cells treated with drug combinations for 48 hours.

Because Tenovin-6 increased hyperacetylation of p53 (Figure 2B), we evaluated whether Tenovin-6 treatment could sensitize ALL cells to the conventional chemotherapeutic agents etoposide and cytarabine. REH and NALM-6 cells were incubated in a serially diluted mixture (at a fixed ratio) of Tenovin-6 and etoposide or cytarabine for 72 hours and then subjected to MTS assay for measurement of cell viability. Synergism was evaluated by the median-effect method of Chou and Talalay [20]. Both the combination of Tenovin-6 and etoposide and the combination of Tenovin-6 and cytarabine synergistically (i.e., combination index < 1) inhibited the viability of ALL cells (Figure 5A). This enhanced effect was further supported by an increase in the proportion of apoptotic cells as evaluated with flow cytometry after Annexin V-FITC/propidium iodide staining and with Western blotting for specific cleavage of PARP, an indicator of apoptosis (Figure 5B and C). Taken together, these data indicated that treatment with Tenovin-6 sensitizes ALL cells to conventional chemotherapeutic agents.

### Tenovin-6 inhibits the Wnt/β-catenin pathway in ALL cells

**Figure 6:**
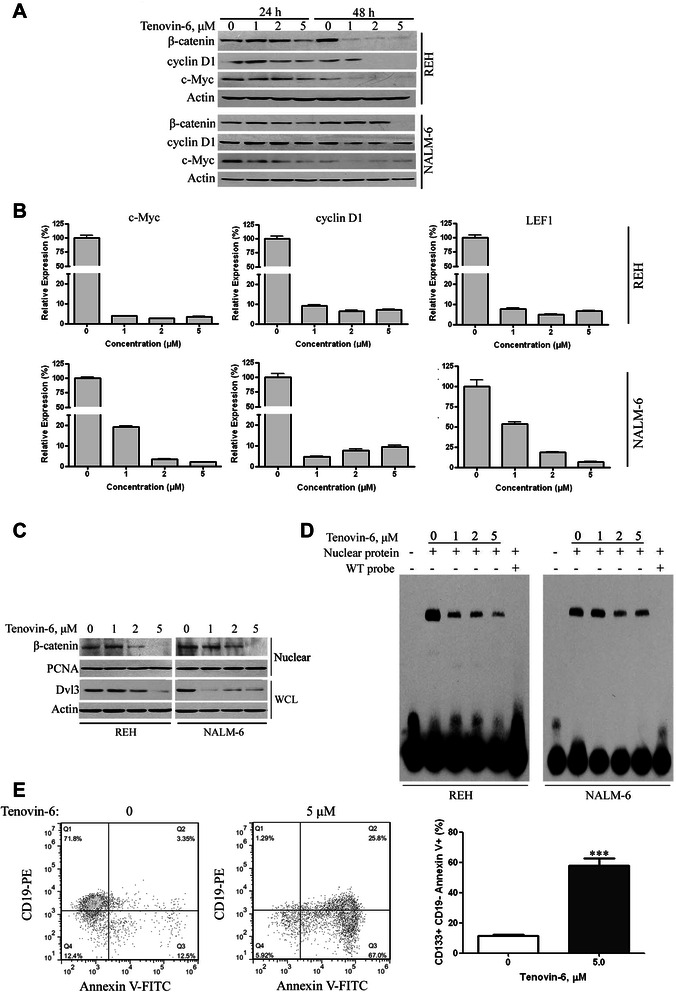
**Tenovin-6 suppresses Wnt/β-catenin signaling in ALL cells and eliminates ALL stem/progenitor cells. A**, REH and NALM-6 cells were treated with various concentrations of Tenovin-6 for 24 and 48 hours. Expression of β-catenin, c-myc, and cyclin D1 was detected by Western blotting with the indicated antibodies. **B**, REH and NALM-6 cells were treated with increasing concentrations of Tenovin-6 for 48 hours, and then qRT-PCR analysis of β-catenin target genes (c-Myc, cyclin D1, and LEF1) was performed using intron-spanning primers. **C**, REH and NALM-6 cells were treated with various concentration of Tenovin-6 for 48 hours. Western blotting for Dvl3 in total cell lysate and β-catenin in nuclear extract was performed. PCNA was used as a nuclear protein reference. **D**, EMSA. Nuclear fractions from REH and NALM-6 cells treated with increasing concentration of Tenovin-6 for 48 hours were incubated with wild-type biotin-labeled TCF probe. Cold competition assay was done using 100-fold unlabeled wild-type probe. **E**, CD133 MicroBeads separating system was applied to bone marrow mononuclear cells of 3 patients with ALL. The sorted CD133^+^ cells were then untreated or treated with 5 μM Tenovin-6 for 48 hours, and apoptosis was evaluated by flow cytometry after cells were stained with Annexin V-FITC and CD19-phycoerythrin.

Canonical Wnt signaling is activated via ligation of Wnt proteins to their respective cell surface receptors Frizzled and LRP6 on HSCs, leading to activation and nuclear translocation of β-catenin, complex formation with TCF, and transcription of target genes. β-catenin is important in the regulation of self-renewal of cancer stem cells [25]. Previous studies showed that SIRT1 is involved in regulation of the Wnt pathway by forming a complex with β-catenin protein and Dishevelled (Dvl) [26]. In addition, p53 was shown to be capable of down-regulating β-catenin level [27]. We therefore asked whether sirtuin inhibition by Tenovin-6 inhibits the Wnt/β-catenin pathway in ALL cells. As anticipated, Tenovin-6 dramatically decreased the total protein level of β-catenin in a time- and concentration-dependent manner (Figure 6A). Because cyclin D1, c-Myc, and LEF1 are known Wnt target genes, we also ascertained the protein levels of these genes [28]. We found that Tenovin-6 treatment suppressed the expression of cyclin D1 and c-Myc (Figure 6A). Decreased expression of the downstream target genes were further proved by real-time qRT-PCR analysis in Tenovin-6-treated ALL cells (Figure 6B).

Because inhibition of SIRT1 could lead to reduction in Dvl proteins [26], we determined whether Tenovin-6 inhibited the protein level of Dvl3 in ALL cells. Western blotting analysis in whole cell lysates revealed that the protein levels of Dvl3 were strikingly lower in both REH and NALM-6 cells treated with Tenovin-6 than in untreated control cells (Figure 6C*, lower 2 lanes*).

We also determined the effect of Tenovin-6 on the β-catenin level in the nuclear fraction, which reflects active β-catenin. Tenovin-6 treatment led to decreased β-catenin levels in the nuclear fractions of ALL cells (Figure 6C, *upper 2 lanes*). Nuclear translocation of β-catenin is required for its functions (i.e., to activate TCF/LEF). Electrophoretic mobility shift assay (EMSA) with TCF/LEF probes revealed a concentration-dependent decrease in nuclear β-catenin in Tenovin-6-treated ALL cells (Figure 6D). Together, these results indicated that Tenovin-6 inhibits Wnt/β-catenin signaling in ALL cells.

### Tenovin-6 eliminates stem/progenitor cells in ALL cells

CD133+/CD19- subfractions from pediatric B-ALL cells are believed to be stem/progenitor cells capable of self-renewing and differentiating into heterogeneous leukemia cells [29]. We next examined the effect of Tenovin-6 on these phenotypically defined stem/progenitor cells from pediatric ALL specimens. With the MACS MicroBead kit, we separated the CD133+ cells from bone marrow mononuclear cells from patients with B-ALL. The purity of the separated cells was confirmed by flow cytometry after incubation with CD133-APC antibody. The sorted CD133+ cells were untreated or treated with 5 μM Tenovin-6 for 48 hours and then subjected to flow cytometry after staining with Annexin V-FITC and CD19-phycoerythrin. Tenovin-6 significantly increased the Annexin V + CD133 + CD19− subpopulation from primary ALL cells (Figure 6E), suggesting that Tenovin-6 treatment eliminates ALL stem/progenitor cells.

## Discussion

Novel targeted therapy for ALL is desperately needed. In the present study, we showed that Tenovin-6, an inhibitor of the class III histone deacetylase sirtuin, was effective as a single agent and in combination with frontline chemotherapeutics against ALL cells. Tenovin-6 treatment activated p53 and induced cell growth inhibition and apoptosis in ALL cell lines and primary ALL cells. Furthermore, we found that Tenovin-6-induced inhibition of SIRT1/2 activity decreased Wnt/β-catenin signaling and eliminated ALL stem/progenitor cells.

SIRT1 deacetylates histone and nonhistone proteins that are involved in many cellular functions. Although the role of SIRT1 in tumorigenesis remains controversial [30-33], SIRT1 expression was shown to be significantly elevated in a number of human cancers, including acute myeloid leukemia [34], prostate cancer [35], colorectal cancer [36], skin squamous cell carcinoma [37], chemoresistant leukemia [38], and CD133-positive glioblastoma stem cells [39]. In accord with these findings, our results showed that the expression of SIRT1 was elevated in primary ALL cells compared with control. Of note, SIRT1 has been demonstrated to promote the development of chronic myelogenous leukemia [18,19].

A number of nonspecific and specific inhibitors of SIRT1 have been discovered, including nicotinamide, sirtinol, splitomicin, HR73, cambinol [32], the tenovins [24], and the indole derivative EX527. Two of these inhibitors, cambinol [32] and tenovin [24], were tested in animal models of cancer and showed great antitumor effect against Burkitt lymphoma and melanoma, respectively. In an *in vitro* peptide deacetylase activity assay, Tenovin-6 was shown to inhibit the activity of SIRT1 and SIRT2 with IC_50_ values of 21 μM and 10 μM, respectively. Our results in the current study demonstrated that treatment of ALL cells with Tenovin-6 at even 1 μM led to hyperacetylation and activation of p53 within approximately 2 to 6 hours.

Results of the present study indicated that Tenovin-6 treatment inhibits growth and induces apoptosis both in ALL cell lines and in primary ALL cells at micromolar concentrations, however, many ALL cells were sensitive to the agents, while several cells were resistant (Figure 3C). We assume that the sensitivity correlates with the p53 mutation status or with the SIRT1/2 expressions, this remains to be further investigated. Of importance, Tenovin-6 is synergistic with the conventional chemotherapeutic agents etoposide and cytarabine and also active against primary cells from patients with relapsed ALL. Tenovin-6 disturbed the cell cycle distribution in ALL cells by restricting the cells in G_1_ phase. The inhibitory effect of Tenovin-6 on cell growth and survival may be explained by the activation of p53 and elevation of p21 after Tenovin-6 treatment.

Cancer stem cells are resistant to chemotherapy and believed to be the source of relapse of tumor. Using phenotypically defined stem/progenitor cells and functional assay, we first showed that Tenovin-6-induced inhibition of SIRT1/2 eliminated ALL stem/progenitor cells. The CD133 + CD19- fraction in ALL cells represents the stem/progenitor cells of ALL. We then found that Tenovin-6 induced marked apoptosis in ALL stem/progenitor cells. Furthermore, Tenovin-6 significantly inhibited the colony-forming capacity of ALL cells (Figure 3D & E).

Tenovin-6-mediated decrease in β-catenin, a key regulator of self-renewal of cancer stem cells may be involved in the elimination of ALL stem/progenitor cells. Our data demonstrated that Tenovin-6 remarkably lowered the levels of total and active β-catenin and blocked the downstream signaling. The underlying mechanism may be associated with Tenovin-6-induced Dvl inhibition and p53 activation. SIRT1 can form a complex with β-catenin and Dvl [26]. Tenovin-6-induced Dvl inhibition is postulated to reduce the stability of the complex. p53 can negatively regulate β-catenin level [40]. Activation of p53 by Tenovin-6 may thus reasonably explain the decrease in β-catenin.

In summary, we found that the novel sirtuin inhibitor Tenovin-6 is effective in killing pre-B ALL cells and eradicating ALL stem/progenitor cells (CD133 + CD19-). Tenovin-6 may represent an important therapeutic agent against pre-B ALL alone or in combination with standard chemotherapeutics and is therefore worthy of further clinical investigation in ALL.

## Conclusions

In the present study, we initially found that the level of SIRT1, a class III histone deacetylase, was higher in primary ALL cells from patients than in peripheral blood mononuclear cells from healthy individuals. we found that the novel sirtuin inhibitor Tenovin-6 is effective in killing pre-B ALL cells and eradicating ALL stem/progenitor cells (CD133 + CD19-). Tenovin-6 may represent an important therapeutic agent against pre-B ALL alone or in combination with standard chemotherapeutics and is therefore worthy of further clinical investigation in ALL.

## Abbreviations

ALL: Acute lymphoblastic leukemia
SIRT1: Histone deacetylase sirtuin 1
qRT-PCR: Reverse transcription and quantitative real-time PCR
RIPA: Radioimmunoprecipitation
EMSA: Electrophoretic mobility shift assay
PARP: Poly(ADP-ribose) polymerase
Dvl: Dishevelled
Annexin V- FITC: Annexin V-fluorescein isothiocyanate
PI: Propidium iodide
